# Effects of Dietary Sulphur Amino Acid Levels on Growth Performance, Meat Quality, Nutrient Digestibility, Serum Biochemistry and Feather Follicle Transcriptomics in Jiangnan White Goslings

**DOI:** 10.3390/ani16121865

**Published:** 2026-06-17

**Authors:** Qingxue Liu, Usman Nazir, Xuan Li, Xiyuan Xing, Xucheng Zheng, Zhi Yang, Haiming Yang, Zhiyue Wang

**Affiliations:** 1 College of Animal Science and Technology, Yangzhou University, Yangzhou 225009, China; 2 College of Bioscience and Biotechnology, Yangzhou University, Yangzhou 225009, China; 3 Joint International Research Laboratory of Agriculture and Agri-Product Safety of Ministry of Education of China, Yangzhou University, Yangzhou 225009, China

**Keywords:** goslings, sulphur-containing amino acids, methionine, meat quality, transcriptomics, feather follicle, finishing period

## Abstract

This study evaluated the effects of dietary sulphur-containing amino acid (SAA) levels provided during the early (1–28 d) and late (29–63 d) growth stages on production traits and feather follicle gene expression in 63-day-old Jiangnan white goslings. A total of 288 one-day-old male goslings were assigned to a 2 × 2 factorial design with two early SAA levels (0.64% and 0.87%) and two late SAA levels (0.62% and 0.74%), which created four treatments: Dd (low–low), Dg (low–high), Gd (high–low), and Gg (high–high). All measurements were performed on 63-day-old birds. Final body weight was significantly affected only by early SAA levels (*p* = 0.03), with the high early groups (Gd and Gg) showing a 3% higher weight (3.99 vs. 3.88 kg) than the low early groups. Late SAA levels had no effect on growth but markedly reduced breast muscle cooking loss (*p* < 0.01) from 26.64% (low late) to 22.70% (high late), which represents a 15% relative improvement in water-holding capacity. A significant early × late interaction (*p* = 0.03) indicated that late high SAA levels partially compensated for early deficiency. High late SAA levels also slightly reduced dry matter digestibility (73.45% → 73.16%, *p* = 0.03), while high early SAA levels increased crude ash digestibility (33.59% → 35.66%, *p* = 0.04). The Dg treatment (low early + high late) caused significantly elevated serum low-density lipoprotein (2.06 vs. 1.42–1.58 mmol/L) and uric acid (348.15 vs. 243.60–294.97 μmol/L), which indicates metabolic stress (*p* < 0.01 and *p* = 0.02 for interaction). Transcriptomic analysis of feather follicles from the Gg and Gd groups (both receiving identical high early SAA supplementation) identified 121 differentially expressed genes, including the downregulation of *MAT1* (cysteine/methionine metabolism) and upregulation of *GHRHR* (neuroactive ligand–receptor interaction) in Gg, providing a molecular basis for improved feather growth. q-PCR validated five selected genes. In conclusion, final body weight is determined by early SAA supply, while late SAA supplementation improves meat juiciness, but abrupt increases from low to high SAA levels cause metabolic disturbances. Under this test conditions, The optimal feeding strategy is 0.87% SAAs (1–28 d) followed by 0.74% SAAs (29–63 d).

## Simple Summary

Sulphur-containing amino acids (methionine and cystine) are essential for the growth and feather development of geese. However, it is not known whether geese need different amounts of these amino acids as they grow from 4 to 9 weeks of age. We fed 288 male Jiangnan white goslings either low or high levels of sulphur amino acids during their first four weeks (1–28 days) and then again during the following five weeks (29–63 days). We measured their body weight, meat quality, nutrient digestibility, blood metabolites and, in a subset of birds, gene expression in feather follicles. The results showed that the amount of sulphur amino acids given in the first four weeks determined the final body weight at 63 days, while the amount given in the later period did not affect weight. However, a higher level of sulphur amino acids in the later period greatly improved meat juiciness by reducing cooking loss by 15%. A combination of low early and high late levels caused metabolic problems (high blood uric acid and LDL cholesterol), so such abrupt changes should be avoided. Analysis of feather follicle genes revealed that high late sulphur amino acids reduced the activity of a gene that breaks down methionine (MAT1) and increased genes that stimulate cell growth (*GHRHR*) in our experimental design, explaining better feather development. The best overall strategy is to give 0.87% sulphur amino acids from 1 to 28 days, followed by 0.74% from 29 to 63 days.

## 1. Introduction

The global goose industry has expanded substantially, with China producing over 70% of the world’s goose meat and more than 600 million birds slaughtered annually. Besides meat, goose feathers and down represent high-value products for the textile industry, making geese a dual-purpose species of significant economic importance [1]. However, goose production still encounters two major challenges: suboptimal growth performance arising from unbalanced dietary formulations; and frequent feather abnormalities such as sparse, brittle feathers and delayed moulting. Both problems are closely linked to nutrition, particularly to the supply of sulphur-containing amino acids (SAAs), namely, methionine and cystine, which play pivotal roles in protein synthesis, methylation reactions, antioxidant defence, and keratin formation [2].

Methionine is the first limiting amino acid in corn–soybean meal-based diets for poultry. It not only participates in protein synthesis but also acts as a methyl donor through S-adenosylmethionine, thereby influencing DNA methylation, gene expression, and lipid metabolism [3]. Moreover, methionine can be converted to cysteine via the transsulphuration pathway. Cysteine and its oxidised dimer, cystine, are the primary building blocks of keratin, the structural protein that constitutes 85–90% of feather mass [4]. The high content of SAAs in feathers—up to 7–13% cystine—reflects their essential role in forming disulfide bonds, which determine feather strength, elasticity and water resistance. Furthermore, through glutathione synthesis, SAAs contribute to the antioxidant defence system, protecting cells from oxidative damage during periods of rapid growth and feather renewal [3,5]. Given these multiple functions, numerous studies have attempted to define the optimal SAA requirements for different goose breeds. For Yangzhou goslings (28–70 d), the optimal dietary methionine level was estimated at about 0.34% (total SAAs around 0.60–0.65%). For Taihu goslings (0–8 weeks), the recommended total SAA range was 0.55–0.65%. For Hungarian goslings (0–6 weeks), 0.60–0.70% methionine produced the best growth and feather quality [6]. In Jilin white geese, increasing methionine from 0.32% to 0.48% improved growth performance and reduced serum uric acid. However, almost all of these studies applied a single, constant SAA level throughout the rearing period [7]. This approach overlooks the fact that nutrient requirements change dynamically as birds grow. The first month of life (brooding period) is characterised by extremely rapid body weight gain and the transition from down feathers to juvenile contour feathers. During this phase, keratin synthesis is particularly intense, making SAA supply critically important. The subsequent period (29–63 d) is marked by continued growth, muscle deposition and final feather maturation. It is likely that the optimal SAA levels for these two stages differ and that both the amount and the timing of SAA supply can influence not only growth but also meat quality and metabolic health. The Jiangnan white goose is a newly developed commercial synthetic line bred by Jiangsu Lihua Animal Husbandry Co., Ltd. [8]. It has gained popularity because of its excellent growth rate, high meat yield and good feather quality. However, stage-specific SAA requirements for this breed have not been established, and the potential carry-over effects of early SAA nutrition on later performance remain unclear. Moreover, the interaction between early and late SAA supply has never been systematically examined in geese. Understanding such interactions is critical for designing practical feeding programmes that can simultaneously optimise growth, meat quality and metabolic well-being while avoiding nutrient waste and metabolic stress [4,9].

Therefore, the primary objective of this study was to evaluate late-stage (29–63 d) SAA levels, as well as their interaction, on growth performance, body measurements, slaughter traits, meat quality, nutrient digestibility and serum biochemistry in 63-day-old Jiangnan white goslings. A secondary objective was to investigate the transcriptomic basis of feather follicle responses to late-stage SAA levels by using RNA sequencing and q-PCR to compare the Gg (high–late) and Gd (low–late) groups that received identical high early SAA supplementation (0.87%). The findings will provide a scientific basis for precision nutrition in the Jiangnan white goose industry.

## 2. Materials and Methods

### 2.1. Ethics Statement

All experimental procedures were approved by the Animal Care and Use Committee of Yangzhou University (Permit No. SYXK (Su) 2021-0027) and were performed in accordance with Chinese animal welfare legislation.

### 2.2. Experimental Design and Diets

A total of 288 healthy 1-day-old male Jiangnan white goslings with similar body weights (98.3 ± 5.2 g) were obtained from Suqian Lihua Animal Husbandry Co., Ltd. (Suqian, China). The experiment used a 2 × 2 factorial design with two early-stage (1–28 d) total SAA levels (0.64% and 0.87%) and two late-stage (29–63 d) total SAA levels (0.62% and 0.74%), which resulted in four treatment combinations: Dd (low early + low late); Dg (low early + high late); Gd (high early + low late); and Gg (high early + high late). Each treatment had six replicates with 11 goslings per replicate.

The corn–soybean meal basal diets were formulated based on previous studies [10,11]. The ingredient composition and nutrient levels of the early-stage diets (1–28 d) and late-stage diets (29–63 d) are presented in Table 1 and Table 2, respectively. All diets were isonitrogenous and isoenergetic within each stage.

### 2.3. Housing and Management

The trial was conducted at the Yangzhou University Gaoyou Experimental Farm (Gaoyou, China). Goslings were housed in wire-floor pens (1.9 m × 1.5 m, 2.85 m^2^ per pen) at a stocking density of 11 birds per pen. Each pen was equipped with two round hanging plastic feeders (30 cm diameter) and two nipple drinkers, providing ad libitum access to feed and water. The poultry house was naturally ventilated with auxiliary exhaust fans to maintain air quality. Pens were cleaned and disinfected before the experiment. Health status was monitored daily, and no treatment-related deaths occurred.

### 2.4. Sample Collection and Measurements (63-Day-Old Birds)

#### 2.4.1. Growth Performance

At 63 d of age, all goslings were weighed after 6 h of feed withdrawal. Feed intake per replicate was recorded throughout the whole experimental period (1–63 d). Average daily gain (ADG), average daily feed intake (ADFI) and feed-to-gain ratio (F/G) were calculated for the entire 1–63 d period.

#### 2.4.2. Slaughter Performance and Organ Indices

The same birds were weighed, then exsanguinated and manually defeathered. Dressing percentage, half-eviscerated yield, eviscerated yield, breast muscle percentage and leg muscle percentage were determined. The heart, liver, muscular stomach (gizzard), glandular stomach (proventriculus), abdominal fat and spleen were removed and weighed to calculate organ indices (organ weight/body weight × 100%).

#### 2.4.3. Meat Quality

Right breast muscle samples were collected. During cutting and trimming, the knife blade was rinsed with chilled water (4 °C) to prevent temperature-induced changes in meat quality. Meat colour (L, a, b) was measured using a CR-400 colorimeter (Konica Minolta, Tokyo, Japan). pH was measured with a pH meter (Testo 205, Lenzkirch, Germany). For cooking loss, muscle samples (2.5 cm thickness) were weighed, placed in sealed plastic bags, heated in a water bath at 80 °C until the core temperature reached 70 °C, then cooled to room temperature in an ice-water bath (0–4 °C) [12], blotted dry and reweighed. Cooking loss (%) = (weight before − weight after)/weight before × 100. Shear force was measured on cooked samples using a texture analyser (TA.XT Plus, Stable Micro Systems, Godalming, UK).

#### 2.4.4. Nutrient Digestibility

At 63 d, one gosling per replicate was placed in a metabolic cage. After a 3-day adaptation period, total excreta were collected for 3 consecutive days. For every 100 g of excreta, 10 mL of 10% hydrochloric acid was added for nitrogen fixation. Excreta samples were dried at 65 °C, equilibrated at room temperature for 24 h, ground and passed through a 40-mesh sieve. Feed samples (early and late diets) and excreta samples were analysed for dry matter, energy, crude protein (Kjeldahl method), crude fat (Soxhlet extraction), crude fibre (filter bag method) and crude ash (furnace incineration) following standard AOAC methods. Acid-insoluble ash (AIA) was used as an indigestible marker. Nutrient digestibility was calculated using the following equation:

Digestibility (%) = 100 − [100 × (AIA in feed/AIA in excreta) × (Nutrient in excreta/Nutrient in feed)]

where AIA = acid-insoluble ash (%). All analyses were performed in duplicate.

#### 2.4.5. Serum Biochemical Parameters

Blood (3 mL) was collected from the wing vein of the same birds before slaughter. After 30 min at room temperature, samples were centrifuged at 2500 rpm for 10 min at 4 °C to obtain serum. Total protein (TP), albumin (ALB), globulin (GLB), albumin/globulin ratio (A/G), triglyceride (TG), high-density lipoprotein (HDL), low-density lipoprotein (LDL) and uric acid (UA) were measured using commercial kits (Nanjing Jiancheng Bioengineering Institute, Nanjing, China), following the manufacturer’s instructions.

#### 2.4.6. Feather Follicle Sampling for Transcriptomics (Gg vs. Gd)

To isolate the effect of late-stage SAA level independent of early-stage variation, feather follicles were collected from the dorsal skin (mid-back area, 4–6 cm anterior to the tail) of birds from the Gg (high–late) and Gd (low–late) groups at 63 d (6 birds per group). Both groups had received identical high early SAA levels (0.87%). Skin samples (2 cm^2^) were collected, the overlying feathers were trimmed, and follicles were dissected under a stereomicroscope, immediately placed in RNA later (Thermo Fisher, Dartford, UK), and stored at −80 °C until analysis.

RNA extraction and sequencing: Total RNA was extracted using QIAzol Lysis Reagent (Qiagen, Hilden, Germany), followed by purification with an RNA Purification Kit (Shanghai Majorbio, Shanghai, China). RNA integrity was assessed using an Agilent 2100 Bioanalyzer (Agilent Technologies, Santa Clara, CA, USA). Libraries were prepared using the Illumina Stranded mRNA Prep Kit (Illumina, San Diego, CA, USA) and sequenced on a NovaSeq 6000 platform (Illumina) with 150 bp paired-end reads.

Read processing and differential expression: Raw reads were filtered to remove adapter sequences, low-quality reads (Q ≤ 20), and reads with >10% N bases. Clean reads were aligned to the Sichuan white goose reference genome (GCF_002166845.1) using HISAT2 (v2.1.0). Gene expression levels were quantified as FPKM using featureCounts (v1.6.3). Differential expression analysis between Gg and Gd was performed using DESeq2 (v1.20.0) with criteria |log_2_ fold change| ≥ 1 and *p* < 0.05.

GO and KEGG enrichment: Gene Ontology (GO) functional enrichment and Kyoto Encyclopedia of Genes and Genomes (KEGG) pathway enrichment analyses were performed using clusterProfiler (v3.8.1), with a threshold of *p* < 0.05.

q-PCR validation: Five differentially expressed genes (RET, CHRM2, IL4I1, AVPR1A, GHRHR) were selected for validation. First-strand cDNA was synthesised using SweScript All-in-One RT SuperMix (Vazyme, Nanjing, China). Real-time PCR was performed on a Bio-Rad CFX96 system (Bio-Rad Laboratories, Hercules, CA, USA) using 2× Universal Blue SYBR Green qPCR Master Mix (Yeasen, Shanghai, China). The cycling programme was: 95 °C for 30 s, followed by 40 cycles of 95 °C for 5 s and 60 °C for 30 s. β-Actin was used as the internal control. Relative expression was calculated using the 2^−ΔΔCt^ method. Primer sequences are listed in Table 3.

### 2.5. Statistical Analysis

All production data were first tested for normality using the Shapiro–Wilk test (*p* > 0.05 considered normal distribution) and for homogeneity of variances using Levene’s test (*p* > 0.05 considered homogeneous). Data met the assumptions of parametric analysis. A two-way analysis of variance (ANOVA) was performed with early-stage SAA level (P-SAA), late-stage SAA level (F-SAA), and their interaction as fixed factors and with replicate pen as the experimental unit. The statistical model was:

Y_ijk_ = μ + α_i_ + β_j_ + (αβ)_ij_ + ε_ijk_

where Y_ijk_ is the dependent variable, μ is the overall mean, α_i_ is the effect of early SAA level (i = 0.64%, 0.87%), β_j_ is the effect of late SAA level (j = 0.62%, 0.74%), (αβ)_ij_ is the interaction term, and ε_ijk_ is the random error.

When a significant main effect or interaction was detected (*p* < 0.05), means were separated using Tukey’s Honestly Significant Difference (HSD) post hoc test. Results are presented as mean ± standard error of the mean (SEM). For each significant effect, F-statistics with degrees of freedom (df) are reported (e.g., F_1,20_ = 6.54, *p* = 0.03). For transcriptomic data, DESeq2 was used with a negative binomial model, and differentially expressed genes were identified using |log_2_ fold change| ≥ 1 and false discovery rate (FDR)-adjusted *p* < 0.05. Differences were considered significant at *p* < 0.05 and highly significant at *p* < 0.01.

## 3. Results

### 3.1. Growth Performance

Table 4 summarises the growth performance of the 63-day-old goslings. Final body weight was significantly influenced only by early-stage SAA level (*p* = 0.03). Birds that had received 0.87% SAAs during the first 28 days (Gd and Gg) had a higher final weight (3.99 kg) 228 than those given 0.64% SAAs (Dd and Dg, 3.88 kg). Neither late-stage SAA level nor the interaction between early- and late-stage SAA levels had any significant effect on final body weight, ADG, ADFI or F/G (*p* > 0.05).

### 3.2. Slaughter Performance and Organ Indices

Table 5 shows slaughter performance. Only leg muscle percentage was significantly influenced by early-stage SAA levels (*p* = 0.02). High early-stage SAA levels (0.87%) increased leg muscle percentage (14.00%) compared to low early-stage levels (12.78%). Dressing percentage, half-eviscerated yield, eviscerated yield and breast muscle percentage were unaffected (*p* > 0.05). Organ indices (heart, liver, muscular stomach, glandular stomach, abdominal fat, spleen) showed no significant differences (Table 6), although spleen index tended to be lower with high late-stage SAA supplementation (*p* = 0.05).

### 3.3. Meat Quality

Table 7 presents breast meat quality. Late-stage SAA level had a highly significant effect on cooking loss (*p* < 0.01). Increasing late-stage SAA levels from 0.62% to 0.74% reduced cooking loss from 26.64% to 22.70%, a relative improvement of approximately 15%. Moreover, a significant interaction between early- and late-stage SAA levels was observed for cooking loss (*p* = 0.03), indicating that the magnitude of the response to late SAA supply depended on early SAA supply. No significant effects were found for pH, shear force or meat colour parameters (L, a, b) at either 0 h or 24 h post-mortem (*p* * > 0.05).

### 3.4. Nutrient Digestibility

As shown in Table 8, late-stage SAA level significantly affected dry matter digestibility (*p* = 0.03). High late-stage SAA levels (0.74%) reduced DM digestibility (73.16%) compared to low late-stage levels (73.45%). Early-stage SAA level significantly affected crude ash digestibility (*p* = 0.04), with high early-stage SAA levels (0.87%) increasing ash digestibility (35.66%) relative to low early-stage levels (33.59%). No other digestibility parameters (energy, crude protein, crude fat, crude fibre) were affected (*p* > 0.05).

### 3.5. Serum Biochemical Parameters

Table 9 shows serum biochemistry at 63 d. Low-density lipoprotein (LDL) and uric acid (UA) were the only parameters showing a significant interaction between early- and late-stage SAA levels (*p* < 0.01 and *p* = 0.02, respectively). The Dg treatment (low early + high late) produced markedly higher LDL (2.06 mmol/L) and uric acid (348.15 μmol/L) compared to the other three treatments. No significant differences were observed for total protein, albumin, globulin, A/G ratio, triglyceride or HDL (*p* > 0.05).

### 3.6. Transcriptomic Analysis of Feather Follicles (Gg vs. Gd)

#### 3.6.1. Differential Expression

**Figure 1:**
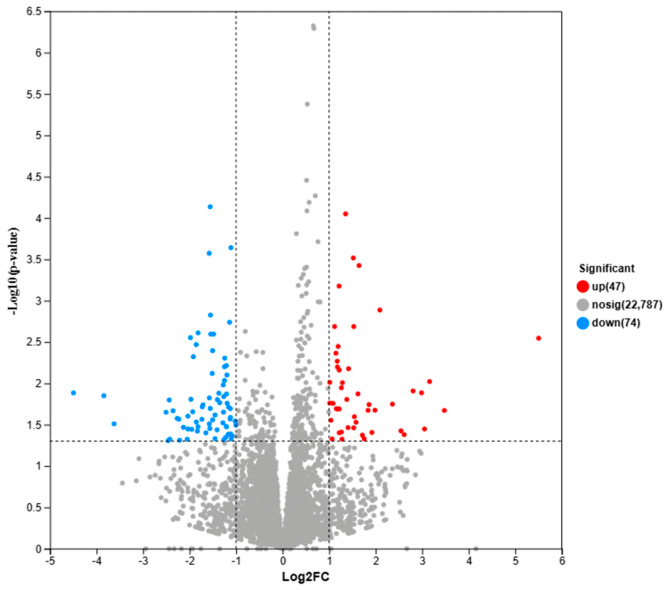
Volcano Plot of Differentially Expressed Genes.

**Figure 2:**
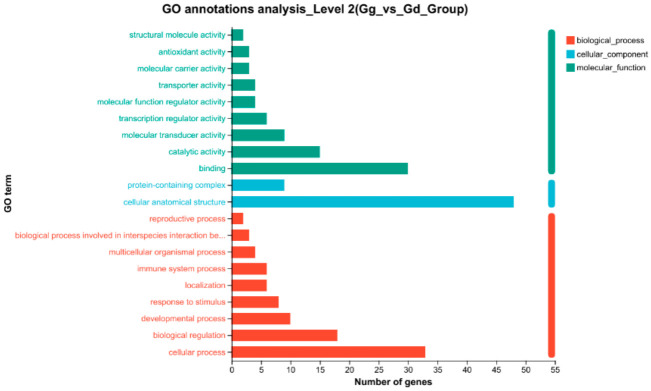
GO analysis of differentially expressed genes.

A total of 221 differentially expressed genes (DEGs) were identified between Gg (high late SAA level) and Gd (low late SAA level), of which 147 were upregulated and 74 were downregulated in Gg (|log_2_FC| ≥ 1, *p* < 0.05) (Figure 1 and Figure 2).

#### 3.6.2. KEGG Pathway Enrichment

KEGG analysis revealed that DEGs were significantly enriched in five pathways (Table 10). Notably, the cysteine and methionine metabolism pathway was downregulated in Gg, involving the gene MAT1 (methionine adenosyltransferase 1). The neuroactive ligand–receptor interaction pathway contained several, including downregulated genes CHRM2 (cholinergic receptor muscarinic 2) and AVPR1A (arginine vasopressin receptor 1A) and upregulated gene GHRHR (growth hormone-releasing hormone receptor).

### 3.7. q-PCR Validation

**Figure 3:**
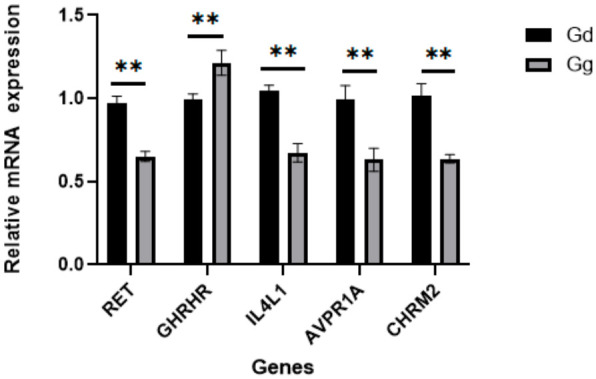
Results of q-PCR validation of differentially expressed genes. **Note:** ** indicates a significant difference between components, *p* < 0.05.

Five selected DEGs (RET, CHRM2, IL4I1, AVPR1A, GHRHR) were validated by q-PCR. The expression trends were consistent with the RNA-seq data, confirming the reliability of the transcriptomic analysis. GHRHR was significantly upregulated in Gg compared to Gd (*p* < 0.05), while RET and IL4I1 were downregulated (Figure 3).

## 4. Discussion

The present study investigated the effects of early and late dietary sulphur-containing amino acid levels on production traits and feather follicle transcriptomics in 63-day-old Jiangnan white goslings. The most striking finding was that final body weight was determined solely by the early-stage SAA level (0.87% vs. 0.64%), with no significant contribution from the late-stage level or its interaction. Birds that received the higher SAA level during the first 28 days weighed, on average, 0.11 kg (about 3%) more at 63 days than those fed the lower level, regardless of whether the late-stage diet contained 0.62% or 0.74% SAAs. This persistent carry-over effect is a clear example of nutritional programming. During the brooding period, the gastrointestinal tract, muscle fibres and somatotropic axis are still developing [13]. Adequate SAA supply in this critical window may permanently increase the number of muscle satellite cells, upregulate growth-related receptors, or alter the set points of the growth hormone–IGF-1 axis [14,15]. Similar long-lasting effects have been reported in broilers, where early methionine restriction reduced myofibre number and subsequent re-feeding could not fully restore growth potential. The absence of a late-stage SAA level effect on weight further suggests that both late levels tested (0.62% and 0.74%) were sufficient to maintain growth, but neither could override the trajectory set earlier [9,16]. From a practical perspective, this underscores the absolute importance of formulating starter diets with adequate SAAs; later adjustments cannot fully compensate for early deficiency [17].

The most commercially significant result was the marked improvement in meat water-holding capacity with high late-stage SAA supplementation. Increasing late SAA levels from 0.62% to 0.74% reduced breast muscle cooking loss from 26.64% to 22.70%, a relative improvement of approximately 15%. Water-holding capacity is a key quality attribute because it directly affects juiciness, texture, and processed meat yield [18]. The mechanism probably involves glutathione, the major intracellular antioxidant, for which methionine and cystine are precursors [19]. Higher SAA availability increases muscle glutathione synthesis, and the elevated glutathione protects muscle cell membranes from oxidative damage during cooking, thereby reducing fluid loss. This interpretation is supported by studies in broilers, where dietary methionine supplementation raised muscle glutathione and decreased cooking loss, and in pigs, where cystine had similar effects [20]. Moreover, a significant early × late interaction was observed for cooking loss. When the early SAA level was low (0.64%), switching to a high late SAA level reduced cooking loss by 6.0 percentage points (Dd → Dg: 28.14% → 22.14%) [21]. When the early SAA level was already high (0.87%), the additional late SAA level produced only a 1.9-point further reduction (Gd → Gg: 25.14% → 23.27%). This pattern reveals a compensatory interaction: muscles retain responsiveness to late SAA levels after a period of early deficiency but with diminishing returns. This is commercially significant because it demonstrates that even if starter diets were suboptimal, feeding a high-SAA finisher can still substantially improve meat quality, although the best results come from consistent adequacy throughout [22].

Regarding nutrient digestibility, high late SAA levels slightly but significantly reduced dry matter digestibility (73.45% → 73.16%, *p* = 0.03). Although the numerical change is small, the consistency across replicates suggests a real biological effect [23]. Possible explanations include increased intestinal osmotic pressure from high free amino acids, shifts in gut microbiota composition (e.g., reduced Lactobacillus abundance), or direct inhibition of digestive enzyme activities [24]. Similar observations have been made in Sichuan white geese and broilers when methionine exceeded requirements [25]. However, the reduction in DM digestibility did not impair growth or feed efficiency, so its practical importance is limited [26]. By contrast, high early SAA levels significantly increased crude ash digestibility (33.59% → 35.66%, *p* = 0.04). This may reflect improved intestinal mucosal integrity and enhanced mineral absorption, possibly through better maintenance of tight junctions. Interestingly, this effect was not seen in the late stage, which indicates a temporary adaptation [27].

The serum biochemistry results revealed a clear warning: the Dg treatment (low early + high late) uniquely produced elevated low-density lipoprotein (2.06 vs. 1.42–1.58 mmol/L) and uric acid (348.15 vs. 243.60–294.97 μmol/L). Uric acid is the end product of amino acid catabolism in birds; high levels indicate inefficient protein utilisation and increased deamination [28]. Elevated LDL suggests disturbed lipid transport. This pattern resembles a metabolic “rebound” phenomenon. After four weeks of relative SAA deficiency, the abrupt increase to 0.74% SAAs may overwhelm the adaptive capacity of the liver and other tissues. The transsulphuration pathway and the methylation cycle may be temporarily unable to handle the sudden influx, leading to accumulation of intermediates (e.g., homocysteine) and increased oxidative stress. Importantly, the Dd (low → low) and Gg (high → high) treatments did not show such abnormalities [29,30]. Therefore, the problem is not high late SAA levels per se but rather the abrupt transition from deficiency to excess. This finding strongly advises against using a “low starter, high finisher” strategy. If cost forces a low SAA starter, the finisher should also be moderate, or the transition should be gradual [31].

The transcriptomic analysis of feather follicles compared Gg (high late) and Gd (low late), both of which received identical high early SAA levels. This design isolated the effect of late SAA level. A total of 221 differentially expressed genes were identified. The downregulation of MAT1 (methionine adenosyltransferase 1) in the cysteine and methionine metabolism pathway is particularly instructive [6]. MAT1 catalyses the conversion of methionine to S-adenosylmethionine, the first step in methionine catabolism [32]. Downregulation of this gene spares methionine from being consumed in catabolic pathways, effectively increasing SAA availability for anabolic uses—most importantly, keratin synthesis. Feathers are composed of β-keratins that are extremely rich in cysteine (7–13%), so shifting SAA partitioning towards keratin formation directly enhances feather growth and quality. This is a novel molecular insight in geese [33,34]. In parallel, the upregulation of CHRM2 (cholinergic receptor muscarinic 2) in the neuroactive ligand–receptor interaction pathway is also notable. Acetylcholine signalling through muscarinic receptors stimulates epithelial cell proliferation in hair follicles and skin appendages. Increased *GHRHR* expression likely activates downstream pathways (e.g., MAPK/ERK) that promote feather germ cell proliferation and accelerate feather growth. Upregulation of AVPR1A and *GHRHR* further suggests enhanced cell survival and GH/IGF-1 signalling, both beneficial for feather follicle activity [33]. Conversely, downregulation of RET in MAPK and calcium pathways may serve as a negative feedback mechanism to prevent excessive proliferation. q-PCR validation of five selected genes confirmed the RNA-seq results, ensuring data reliability [34,35,36]. Collectively, the transcriptomic data show that a high late SAA level acts at two levels: reducing SAA catabolism to provide more building blocks for keratin, and activating proliferative signalling in feather follicles to accelerate feather growth.

In summary, this study provides robust evidence that early SAA supply programmes final body weight, whereas late SAA supplementation primarily improves meat water-holding capacity. Abrupt increases from low to high SAAs cause metabolic stress and should be avoided. The Gg treatment (0.87% SAAs, 1–28 d; 0.74% SAAs, 29–63 d) is the optimal feeding strategy, delivering the highest body weight, excellent meat juiciness, normal serum metabolites, and a favourable feather follicle transcriptome. However, this study has limitations: transcriptomic analysis was performed only at the late stage, and protein validation of MAT1 was lacking, while feather quality traits were not phenotyped. Future research should integrate longitudinal transcriptomics with proteomics, quantify feather structural properties, and measure muscle glutathione and hepatic oxidative stress markers to establish causal mechanisms linking SAA nutrition to meat juiciness and metabolic health. Functional validation of *MAT1* and *GHRHR* via targeted gene manipulation will further solidify the molecular basis of SAA partitioning in geese.

## 5. Conclusions

In conclusion, the final body weight of 63 day old Jiangnan white goslings is determined solely by the total sulphur-containing amino acid level fed during the first 28 days, with high early SAA levels (0.87%) providing a lasting 3% weight advantage over low early SAA levels (0.64%), while late stage SAA level (29–63 d) does not affect growth but significantly improves meat quality by reducing breast muscle cooking loss by approximately 15% when increased from 0.62% to 0.74%, thereby enhancing juiciness and water holding capacity through a compensatory interaction that partially corrects early deficiency. The Dg treatment (low early + high late) causes metabolic stress manifested as elevated serum LDL and uric acid, which indicates that abrupt increases from low to high SAA levels should be avoided. Transcriptomic analysis of feather follicles reveals that a high late SAA level downregulates *MAT1* in the cysteine/methionine metabolism pathway, sparing SAAs for keratin synthesis, and upregulates *GHRHR* in the neuroactive ligand receptor interaction pathway, stimulating follicular cell proliferation, thus providing a molecular basis for improved feather growth. Therefore, the optimal feeding strategy for Jiangnan white goslings is 0.87% total SAAs from 1 to 28 days followed by 0.74% total SAAs from 29 to 63 days (Gg treatment). Importantly, these recommended SAA values are expressed on a total basis rather than a digestible basis and may vary depending on the methionine digestibility coefficients of the feed ingredients used in practical diets.

## Figures and Tables

**Table 1:** Composition and nutrient levels of early-stage (1–28 d) diets (air-dried basis, %).

Item	Low SAAs (0.64%)	High SAAs (0.87%)
Ingredients		
Corn	61.23	61.21
Soybean meal	26.93	26.83
Rice husk	2.76	2.76
Wheat bran	4.91	4.91
Limestone	1.05	1.05
Calcium hydrogen phosphate	1.60	1.60
Salt	0.30	0.30
L-Lysine	0.10	0.10
DL-Methionine	0.12	0.20
L-Cystine	0.00	0.04
Premix ^1^	1.00	1.00
Total	100.00	100.00
Nutrient levels ^2^		
ME (MJ/kg)	11.32	11.31
Crude protein	17.48	17.55
Calcium	0.80	0.80
Total phosphorus	0.76	0.74
Crude fibre	4.27	4.27
Lysine	0.97	0.97
Methionine	0.40	0.48
Cysteine + cystine	0.24	0.39
Total SAA	0.64	0.87

^1^ Premix supplied per kg diet: VA 12,000 IU; VD_3_ 4000 IU; VE 18 IU; VK 1.5 mg; VB_1_ 0.90 mg; VB_2_ 8 mg; VB_6_ 3.2 mg; VB_12_ 0.01 mg; niacin 40 mg; pantothenic acid 11 mg; folic acid 0.65 mg; choline 0.45 g; biotin 0.05 mg; Fe 0.06 g; Cu 0.01 g; Mn 0.095 g; Zn 9 g; I 50 mg; Se 0.03 mg. ^2^ ME, calcium, total phosphorus and lysine are calculated values; others are measured.

**Table 2:** Composition and nutrient levels of late-stage (29–63 d) diets (air-dried basis, %).

Item	Low SAAs (0.62%)	High SAAs (0.74%)
Ingredients		
Corn	60.25	60.30
Soybean meal	23.65	23.30
Rice husk	7.75	7.78
Wheat bran	4.26	4.35
Limestone	1.00	1.00
Calcium hydrogen phosphate	1.65	1.65
Salt	0.30	0.30
L-Lysine	0.10	0.10
DL-Methionine	0.09	0.17
L-Cystine	0.00	0.05
Premix ^1^	1.00	1.00
Total	100.00	100.00
Nutrient levels ^2^		
ME (MJ/kg)	11.01	11.00
Crude protein	16.01	16.00
Calcium	0.82	0.82
Total phosphorus	0.64	0.63
Crude fibre	5.98	6.00
Lysine	0.96	0.96
Methionine	0.35	0.42
Cysteine + cystine	0.27	0.33
Total SAA	0.62	0.74

^1^ Premix supplied per kg diet: VA 12,000 IU; VD_3_ 4000 IU; VE 18 IU; VK 1.5 mg; VB_1_ 0.90 mg; VB_2_ 8 mg; VB_6_ 3.2 mg; VB_12_ 0.01 mg; niacin 40 mg; pantothenic acid 11 mg; folic acid 0.65 mg; choline 0.45 g; biotin 0.05 mg; Fe 0.06 g; Cu 0.01 g; Mn 0.095 g; Zn 9 g; I 50 mg; Se 0.03 mg. ^2^ ME, calcium, total phosphorus and lysine are calculated values; others are measured.

**Table 3:** Primer sequences for the genes utilised in the qRT-PCR.

Gene	Forward Primer (5′ → 3′)	Reverse Primer (5′ → 3′)	Accession No.
*RET*	GCTGGACATCGACCTCAACG	GATGGTGAAGCTGCTGCTGA	XM_047834623.1
*CHRM2*	TCCTGCTGCTCATCGTCATC	GATGATGACGAAGCCGAGGA	XM_013196779.2
*IL4I1*	GCTGGAGATGCTGAAGACCA	CCGATGATGAAGCTGCTGGT	XM_047829876.1
*AVPR1A*	GCTGCTGATGCTGACCTCAT	CGATGATGACGAAGCCGAAG	XM_047823456.1
*GHRHR*	TCTGGTGGCCTGGCTTGGA	GGGATCGTGAGGACTGGGAG	XM_013195521.1
β-actin	ATGCCAGGGTACATTGTGGTA	TGTCATCTTCTCACGGTTGGC	XM_013185313.2

**Table 4:** Growth performance of 63-day-old goslings (1–63 d).

Treatment	P-SAA (%)	F-SAA (%)	Body Weight (kg)	ADG (g)	ADFI (g)	F/G (g/g)
Dd	0.64	0.62	3.93	66.59	287.31	4.32
Dg	0.64	0.74	3.82	69.77	281.23	4.04
Gd	0.87	0.62	3.98	67.45	280.19	4.16
Gg	0.87	0.74	4.00	67.76	275.93	4.08
SEM			0.09	0.38	0.40	0.21
Main effects						
P-SAA 0.64%			3.88	68.18	284.27	4.18
P-SAA 0.87%			3.99	67.61	278.06	4.12
F-SAA 0.62%			3.98	67.02	283.75	4.24
F-SAA 0.74%			4.01	68.77	278.58	4.06
SEM			0.09	0.55	0.81	0.01
*p*-values						
P-SAA			0.03	0.66	0.19	0.53
F-SAA			0.48	0.19	0.27	0.07
Interaction			0.21	0.28	0.84	0.32

P-SAA, early-stage SAA level; F-SAA, late-stage SAA level; ADG, average daily gain; ADFI, average daily feed intake; F/G, feed-to-gain ratio; SEM, standard error of the mean. n = 6 per treatment.

**Table 5:** Slaughter performance of 63-day-old goslings (%).

Treatment	Dressing %	Half-Eviscerated %	Eviscerated %	Breast Muscle %	Leg Muscle %
Dd	88.16	81.22	72.64	8.85	13.18
Dg	88.31	81.35	72.46	7.59	12.39
Gd	88.28	81.06	72.47	8.39	14.00
Gg	89.20	82.49	73.89	8.71	14.00
SEM	0.98	1.39	1.05	1.44	2.65
Main effects					
P-SAA 0.64%	88.23	81.28	72.55	8.22	12.78
P-SAA 0.87%	88.74	81.78	73.17	8.55	14.00
F-SAA 0.62%	88.22	81.14	72.54	8.62	13.59
F-SAA 0.74%	88.75	81.92	73.18	8.15	13.20
SEM	0.79	0.99	1.19	2.01	0.91
*p*-values					
P-SAA	0.31	0.28	0.38	0.49	0.02
F-SAA	0.29	0.18	0.36	0.33	0.42
Interaction	0.43	0.26	0.25	0.11	0.42

P-SAA, early-stage sulphur amino acid level; F-SAA, late-stage sulphur amino acid level; SEM, standard error of the mean. n = 6 per treatment.

**Table 6:** Organ indices of 63-day-old goslings (1–63 d) (%).

Treatment	Heart	Liver	Muscular Stomach	Glandular Stomach	Abdominal Fat	Spleen
Dd	0.60	2.02	3.41	0.36	2.18	0.10
Dg	0.57	2.01	3.39	0.38	2.54	0.09
Gd	0.65	2.10	3.55	0.38	1.95	0.11
Gg	0.63	1.94	3.48	0.36	2.19	0.07
SEM	0.40	0.65	0.87	0.75	0.45	0.13
Main effects						
P-SAA 0.64%	0.59	2.02	3.40	0.37	2.36	0.10
P-SAA 0.87%	0.64	2.02	3.52	0.37	2.07	0.09
F-SAA 0.62%	0.62	2.06	3.48	0.37	2.06	0.10
F-SAA 0.74%	0.60	1.98	3.44	0.37	2.36	0.08
SEM	0.40	0.65	0.87	0.75	0.45	0.13
*p*-values						
P-SAA	0.13	1.00	0.46	0.90	0.27	0.87
F-SAA	0.45	0.34	0.74	0.96	0.25	0.05
Interaction	0.93	0.41	0.92	0.29	0.82	0.29

P-SAA, early-stage sulphur amino acid level; F-SAA, late-stage sulphur amino acid level; SEM, standard error of the mean. n = 6 per treatment.

**Table 7:** Breast meat quality of 63-day-old goslings.

Treatment	pH_0_	pH_24_	Shear Force (N)	Cooking Loss (%)
Dd	7.65	6.38	60.21	28.14 ^a^
Dg	7.62	6.12	60.25	22.14 ^c^
Gd	7.83	5.90	61.24	25.14 ^b^
Gg	7.70	6.33	56.28	23.27 ^bc^
SEM	0.11	0.93	0.38	0.83
Main effects				
P-SAA 0.64%	7.63	6.25	60.23	25.14
P-SAA 0.87%	7.77	6.12	58.76	24.20
F-SAA 0.62%	7.74	6.14	60.72	26.64
F-SAA 0.74%	7.66	6.23	58.26	22.70
SEM	0.12	1.10	0.98	1.20
*p*-values				
P-SAA	0.64	0.57	0.68	0.30
F-SAA	0.77	0.72	0.50	<0.01
Interaction	0.86	0.14	0.49	0.03

P-SAA, early-stage sulphur amino acid level; F-SAA, late-stage sulphur amino acid level; SEM, standard error of the mean, ^abc^ Different superscripts within a column indicate significant difference. n = 6 per treatment.

**Table 8:** Nutrient digestibility in 63-day-old goslings (%).

Treatment	Dry Matter	Energy	Crude Protein	Crude Fat	Crude Fiber	Crude Ash
Dd	74.53	79.75	51.31	84.31	27.29	33.05
Dg	74.12	78.65	48.62	85.12	26.85	34.12
Gd	72.36	78.72	51.12	86.36	27.12	36.56
Gg	72.20	77.56	49.63	87.21	25.31	34.75
SEM	0.32	0.24	1.13	0.85	0.42	0.21
Main effects						
P-SAA 0.64%	74.33	79.20	49.97	84.72	27.07	33.59
P-SAA 0.87%	72.28	78.14	50.38	86.79	26.22	35.66
F-SAA 0.62%	73.45	79.24	51.22	85.34	27.21	34.81
F-SAA 0.74%	73.16	78.11	49.13	86.17	26.08	34.44
SEM	0.44	0.56	1.09	1.22	0.97	0.62
*p*-values						
P-SAA	0.65	0.19	0.64	0.56	0.53	0.04
F-SAA	0.03	0.56	0.31	0.09	0.09	0.61
Interaction	0.72	0.53	0.61	0.32	0.34	0.56

P-SAA, early-stage sulphur amino acid level; F-SAA, late-stage sulphur amino acid level; SEM, standard error of the mean. n = 6 per treatment.

**Table 9:** Serum biochemical parameters of 63-day-old goslings.

Treatment	TP (g/L)	ALB (g/L)	GLB (g/L)	A/G	TG (mmol/L)	HDL (mmol/L)	LDL (mmol/L)	Uric Acid (μmol/L)
Dd	44.28	16.02	28.27	0.58	0.83	2.27	1.42 ^b^	259.90 ^b^
Dg	44.28	16.08	28.20	0.57	1.28	2.27	2.06 ^a^	348.15 ^a^
Gd	42.45	15.47	26.98	0.57	1.01	2.22	1.58 ^b^	294.97 ^ab^
Gg	42.27	16.13	26.13	0.62	1.07	2.43	1.58 ^b^	243.60 ^b^
SEM	0.33	0.30	0.72	0.42	0.12	0.78	0.01	0.06
Main effects								
P-SAA 0.64%	44.28	16.05	28.23	0.57	1.06	2.27	1.74	304.03
P-SAA 0.87%	44.36	15.80	26.56	0.60	1.04	2.32	1.58	269.28
F-SAA 0.62%	43.37	15.74	27.63	0.57	0.92	2.25	1.50	277.43
F-SAA 0.74%	43.28	16.11	27.17	0.60	1.17	2.35	1.82	295.85
SEM	0.43	0.31	0.61	0.01	0.03	0.12	0.11	0.91
*p*-values								
P-SAA	0.33	0.66	0.29	0.39	0.90	0.74	0.11	0.21
F-SAA	0.96	0.51	0.77	0.39	0.06	0.49	<0.01	0.50
Interaction	0.96	0.60	0.81	0.25	0.13	0.49	<0.01	0.02

TP, total protein; ALB, albumin; GLB, globulin; A/G, albumin-to-globulin ratio; TG, triglyceride; HDL, high-density lipoprotein; LDL, low-density lipoprotein;; P-SAA, early-stage sulphur amino acid level; F-SAA, late-stage sulphur amino acid level; SEM, standard error of the mean. ^ab^ Different superscripts within a column indicate significant difference (*p* < 0.05). n = 6 per treatment.

**Table 10:** KEGG pathways enriched in feather follicles of Gg vs. Gd.

Pathway	Number of DEGs	Direction	Key Genes
Cysteine and methionine metabolism	1	Down	MAT1
Neuroactive ligand–receptor interaction	6	Mixed (4 up, 2 down)	CHRM2 (up), AVPR1A (up), GHRHR (up)
MAPK signalling pathway	1	Down	RET
Calcium signalling pathway	4	Down	RET, CHRM2, DRD5, AVPR1A
Protein digestion and absorption	1	Up	LOC106029972

## Data Availability

The raw data supporting the conclusions of this article will be made available by the authors upon request.
